# Interaction of Proliferation Cell Nuclear Antigen (PCNA) with c-Abl in Cell Proliferation and Response to DNA Damages in Breast Cancer

**DOI:** 10.1371/journal.pone.0029416

**Published:** 2012-01-04

**Authors:** Huajun Zhao, Po-Chun Ho, Yuan-Hung Lo, Alexsandra Espejo, Mark T. Bedford, Mien-Chie Hung, Shao-Chun Wang

**Affiliations:** 1 Department of Cancer and Cell Biology, University of Cincinnati College of Medicine, Cincinnati, Ohio, United States of America; 2 Department of Molecular Carcinogenesis, M. D. Anderson Cancer Center, University of Texas, Smithville, Texas, United States of America; 3 Department of Molecular and Cellular Oncology, M. D. Anderson Cancer Center, University of Texas, Houston, Texas, United States of America; 4 Center for Molecular Medicine, China Medical University and Hospital, Taichung, Taiwan; 5 Graduate Institute of Cancer Biology, China Medical University and Hospital, Taichung, Taiwan; Wayne State University School of Medicine, United States of America

## Abstract

Cell proliferation in primary and metastatic tumors is a fundamental characteristic of advanced breast cancer. Further understanding of the mechanism underlying enhanced cell growth will be important in identifying novel prognostic markers and therapeutic targets. Here we demonstrated that tyrosine phosphorylation of the proliferating cell nuclear antigen (PCNA) is a critical event in growth regulation of breast cancer cells. We found that phosphorylation of PCNA at tyrosine 211 (Y211) enhanced its association with the non-receptor tyrosine kinase c-Abl. We further demonstrated that c-Abl facilitates chromatin association of PCNA and is required for nuclear foci formation of PCNA in cells stressed by DNA damage as well as in unperturbed cells. Targeting Y211 phosphorylation of PCNA with a cell-permeable peptide inhibited the phosphorylation and reduced the PCNA-Abl interaction. These results show that PCNA signal transduction has an important impact on the growth regulation of breast cancer cells.

## Introduction

The c-Abl proto-oncogene is a multi-functional non-receptor tyrosine kinase that shuttles between the cytoplasm and the nucleus [1]. A substantial body of knowledge has been established regarding the mechanisms of c-Abl in regulating cell migration, the response to oxidative stresses, and apoptosis [2]. The c-Abl kinase was originally identified as a frequent target of oncogenic chromosomal translocation in hematopoietic neoplasia, but has been increasingly recognized for its involvement in solid tumors. In lung and breast cancers, deregulated c-Abl kinase contributes to tumor development [2]–[5]. In breast cancer, activated c-Abl kinase promotes cancer progression [4], [6], while inhibition of c-Abl blocks the transforming phenotypes by suppressing anchorage-independent growth, inducing apoptosis, and inhibiting cell proliferation [5]. Consistent with this, our recent study showed that increased c-Abl expression is a frequent event in breast cancer (∼40%) [7].

Proliferating cell nuclear antigen (PCNA) is the molecular coordinator in the core DNA synthesis machinery [8]–[16]. PCNA forms a homotrimeric ring encircling the DNA double helix and acts as a molecular platform to recruit proteins involved in DNA synthesis, cell-cycle control, and DNA-damage response and repair [8], [13], [16]–[18]. PCNA exists in two distinct forms: the replication-competent chromatin-bound form and the chromatin-unbound form, which is not involved in DNA synthesis [19]. Not much is known about how the two populations of PCNA are regulated. We previously reported that the chromatin-bound PCNA, but not the unbound form, was phosphorylated at Y211 (phospho-Y211) by the EGF receptor [20]. This phosphorylation event was upregulated during the S phase of the cell cycle. Further study demonstrated that this phosphorylation enhanced the stability of chromatin-associated PCNA and enhanced its activity in DNA replication.

In the current study, we identify c-Abl as a binding partner of PCNA and show that Y211 phosphorylation of PCNA serves as a binding signal for PCNA to associate with c-Abl under normal growth conditions and in cells responding to DNA-damage stresses. We further demonstrate that c-Abl promotes chromatin association of PCNA and that Y211 phosphorylation is an important cell growth-related event downstream of c-Abl.

## Results

**Figure 1:**
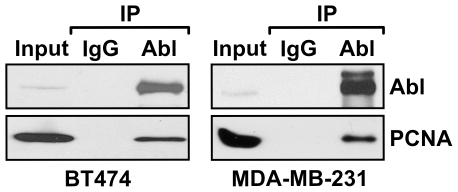
PCNA interacts with c-Abl in vitro and in vivo. Endogenous interaction of PCNA with c-Abl. Interaction of endogenous PCNA and c-Abl in breast cancer cell lines BT474 cells (left panel) and MDA-MB-231 cells (right panel) demonstrated by immunoprecipitation with an anti–c-Abl antibody. After gel separation, the co-precipitated PCNA and c-Abl were detected by using the corresponding antibodies.

We tested whether Y211 phosphorylation of PCNA can serve as a signaling event that, in turn, regulates its function. To do this, we screened a microarray of functional domains derived from different proteins and tested whether phospho-Y211 preferentially associated with these motifs. Synthetic peptides, encompassing the wild-type sequence surrounding the Y211 residue or the same peptide with phosphorylated Y211, were conjugated to a fluorescent dye (Cy3) and used for probing the microarray (data not shown). The Cy3-conjugated peptide with the non-phosphorylated wild-type sequence did not bind to any of the functional domains. In contrast, incubation with the phosphorylated peptide identified the SH2 domain of the non-receptor tyrosine kinase c-Abl as a phospho-Y211-binding motif. Further verification with co-immunoprecipitation (co-IP) using extracts of MDA-MB-231 and BT474 cells demonstrated that PCNA and c-Abl formed a complex in vivo (**Fig. 1**).

**Figure 2:**
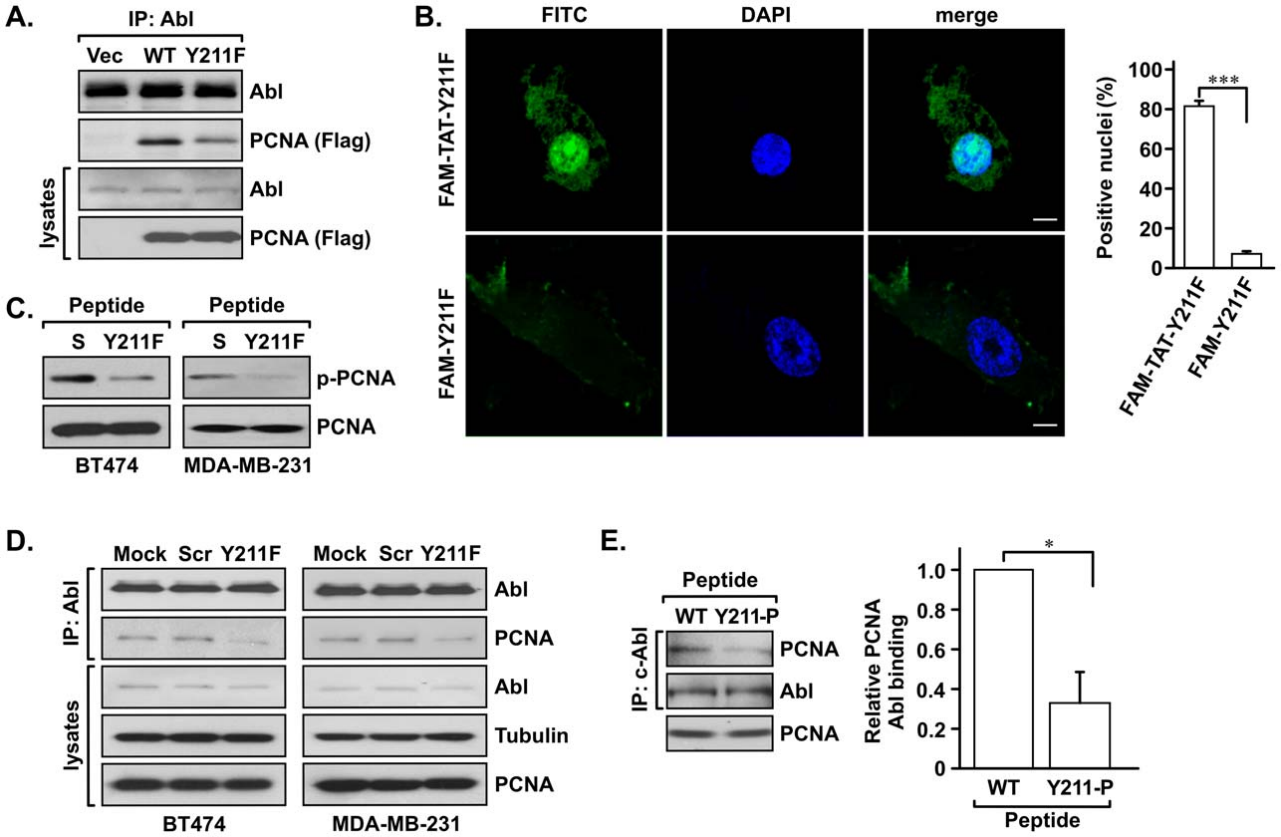
Y211 phosphorylation of PCNA is important for c-Abl association. **A.** HEK293T cells were transfected with FLAG-tagged wild-type or Y211F PCNA. Endogenous c-Abl was then immunoprecipitated with an anti-c-Abl antibody. The levels of co-immunoprecipitated FLAG-PCNA were determined by western analysis. **B.** Left panel, representative images of MDA-MB-231 cells treated for 30 min with 7.5 µM of the indicated FAM-labeled synthetic peptide with (FAM-TAT-Y211F) or without (FAM-Y211F) the TAT sequence conjugation. Localization of the peptides was determined by fluorescence confocal microscopy. Scale bar = 5 µm. Right panel, the numbers of cells with positive nucleus were counted in three independent fields for each treatment and the data are plotted. ***, P<0.005. **C.** The Y211F peptide inhibited Y211 phosphorylation of PCNA in breast cancer cells. The breast cancer cell lines BT474 and MDA-MB-231 were treated with 15 µM of the Y211F peptide or the control scramble peptide for 12 h. Cells were then lysed and phospho-Y211 PCNA was immunoprecipitated by using the anti-phospho–Y211 antibody, which was then detected by western blotting with an anti-PCNA antibody. The levels of input PCNA in the lysates are shown in the lower panel. **D.** The Y211F peptide inhibited the interaction between PCNA and c-Abl. Cells were treated with the peptides as described in C, and the cell lysates were then immunoprecipitated with an anti–c-Abl antibody. The co-precipitated PCNA and c-Abl were then examined by western analysis. **E.** Left panel, phospho-Y211 peptide inhibits the interaction between PCNA and c-Abl. BT474 cells were treated with the TAT-conjugated Y211 peptide with (Y211-p) or without (WT) phosphorylation at the Y211 residue. Cell lysates were immunoprecipitated with an anti-c-Abl antibody. The immune-complexes were analyzed with western blotting for the levels of interacting PCNA. Right panel, the data of three independent repeats were quantitated and plotted. *, P<0.05.

These results indicate that PCNA interacts with c-Abl, and suggest that the interaction is mediated by Y211 phosphorylation. To further test the importance of Y211 phosphorylation in the Abl-PCNA interaction, wild-type PCNA or mutant PCNA in which the Y211 residue was replaced with a phenylalanine (Y211F) was transfected into HEK293T cells. Expression of the transfected PCNA was at corresponding levels relative to the endogenous PCNA (**Figure S1**). Endogenous c-Abl was immunoprecipitated with a c-Abl–specific antibody and the level of co-precipitated ectopic PCNA was determined by western analysis. As shown in **Figure 2A**, binding between the Y211F mutant and c-Abl was reduced compared with that of wild-type PCNA, suggesting that phosphorylation of Y211 is important for the association between PCNA and c-Abl. To further define the role of Y211 phosphorylation of PCNA in association with c-Abl, a synthetic peptide containing the sequence of the proximal region of Y211 in which the Y211 residue was replaced with phenylalanine was used to block phosphorylation at Y211 (hereafter referred to as the Y211F peptide) [20]. As a negative control, we used a peptide with the same amino acid residues as the Y211F peptide but in a scrambled order (referred to hereafter as the scramble peptide). These peptides were conjugated with the HIV-derived TAT sequence at the N terminus, which was capable of transducing the peptides to the nuclear compartment [21]. This was demonstrated by the clear nuclear localization of 5(6)-carboxyfluorescein (FAM)-labeled Y211F peptide (comparing FAM-TAT-Y211F and FAM-Y211F in **Fig. 2B**). Importantly, treatment with the Y211F peptide, but not with the same dose of the control scramble peptide, resulted in inhibition of Y211 phosphorylation of PCNA in BT474 and MDA-MB-231 cells (**Fig. 2C**), and blocked the interaction between endogenous PCNA and c-Abl proteins in both cell lines (**Fig. 2D**). The importance of Y211 phosphorylation for c-Abl interaction was further supported by incubating cells with synthetic peptides of the wild-type sequence with and without phosphorylation at the Y211 residue (**Fig. 2E**). The results showed that the phosphorylated Y211 peptide was more potent in blocking the Abl-PCNA interaction than the non-phosphorylated counterpart. Taken together, these results indicate that phosphorylation of Y211 on PCNA is critical for the interaction between PCNA and c-Abl.

**Figure 3:**
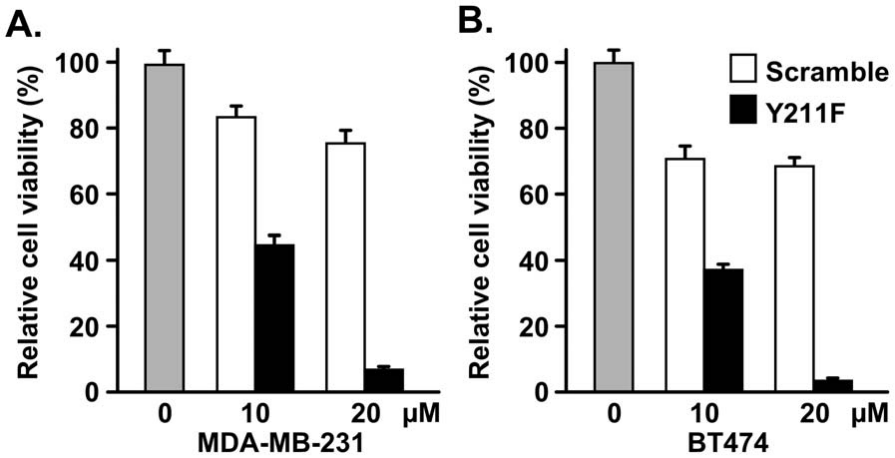
Y211 phosphorylation is an important growth signal downstream of c-Abl. The breast cancer cell lines MDA-MB-231 (A) and BT474 (B) were treated with the Y211F peptide or the control scramble peptide at the indicated doses for 48 hours. Surviving cells were then quantified by MTT assay and the results were plotted.

**Figure 4:**
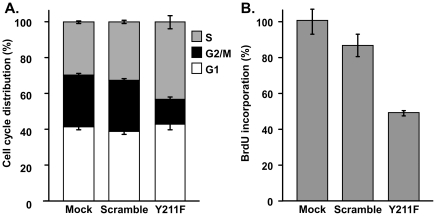
Targeting breast cancer cell line MDA-MB-231 with the Y211F peptide resulted in growth inhibition. **A.** Cells mock-treated with vehicle alone or treated with the scramble or Y211F peptide (15 µM) were subjected to flow cytometry analysis. The percentages of cells in the G1, S, and G2/M phases were plotted. **B.** DNA synthesis activity in the treated cells was determined by a colorimetric BrdU-incorporation analysis. For each data point, the amount of incorporated BrdU was normalized to the percentage of viable cells, as determined by a side-by-side MTT assay.

**Figure 5:**
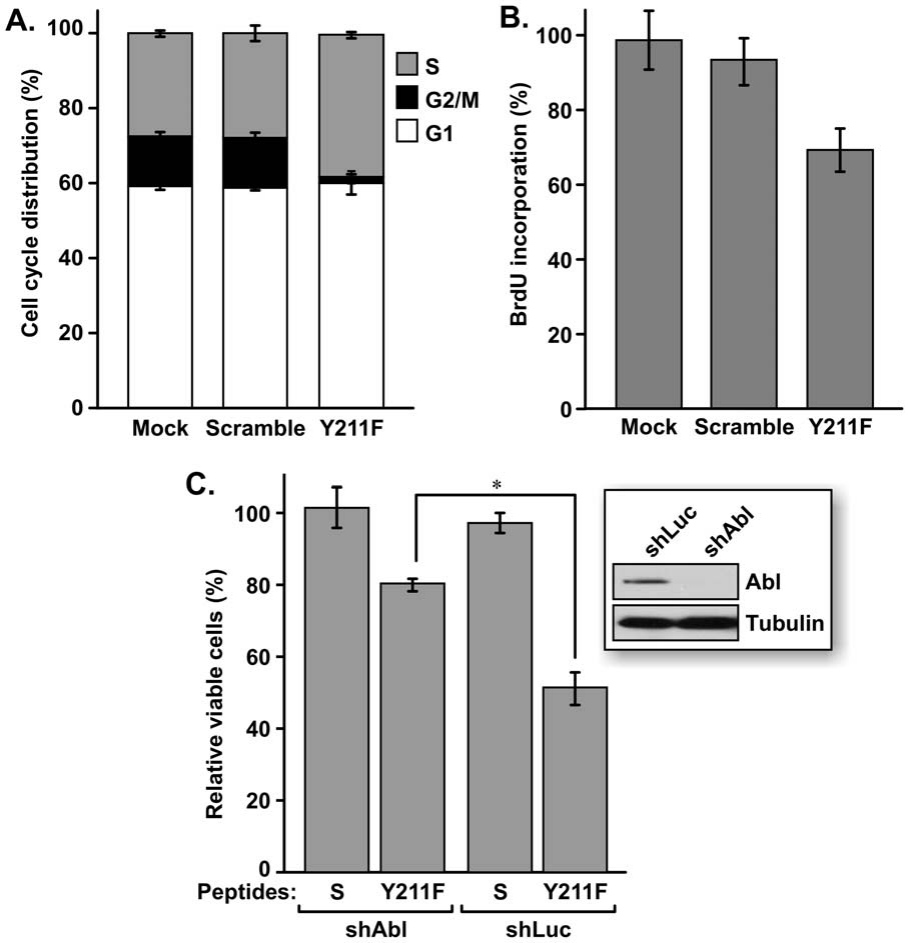
Growth inhibition of BT474 cells by targeting Y211 phosphorylation of PCNA. **A.** Cells mock-treated with vehicle alone or treated with the scramble or Y211F peptides (15 µM) were subjected to flow cytometry analysis. The percentages of cells in the G1, S, and G2/M phases were plotted. **B.** DNA synthesis activity in the treated cells was determined by a colorimetric BrdU-incorporation analysis. For each data point, the amount of incorporated BrdU was normalized to the percentage of viable cells, as determined by a side-by-side MTT assay. **C.** Depletion of c-Abl decreases sensitivity to Y211F peptide-mediated growth inhibition. A derivative of BT474 cells harboring an shRNA against c-Abl (BT474/shAbl ) or luciferase (BT474/shLuc) were treated with 10 µM Y211F peptide or the scramble peptide for 48 h. Surviving cells were then assessed by MTT assay and the results were plotted. *, P<0.05.

To test the impact of inhibiting Y211 phosphorylation of PCNA in breast cancer cell growth, MDA-MB-231 (**Fig. 3A**) and BT474 (**Fig. 3B**) cells were treated with the Y211F peptide or the control peptide. Treatment with the Y211F peptide inhibited growth in both cell lines in a dose-dependent manner, indicating that Y211 phosphorylation is a critical event in breast cancer cells. Indeed, targeting Y211 phosphorylation by the Y211F peptide suppressed proliferation of MDA-MB-231 (**Fig. 4**) and BT474 (**Fig. 5**) cells. Flow cytometry analysis showed that MDA-MB-231 cells treated with the Y211F peptide revealed a significantly increased proportion of cells in the S phase and decreased the proportion in the G2-M phase of the cell cycle, while cells treated with the control scramble peptide had cell cycle distributions similar to the mock-treated cells (**Fig. 4A**). The cells accumulated in the S phase were not engaged in DNA synthesis. This was revealed by BrdU incorporation analysis showing that treatment with the Y211F peptide resulted in reduced DNA synthesis activity per viable cell (**Fig. 4B**). Similar effects were observed in BT474 cells (**Fig. 5**). These results suggest that Y211 phosphorylation of PCNA conveys a growth-promoting function partly through its interaction with c-Abl. To further determine whether Y211 phosphorylation cooperates with c-Abl in enhancing cell growth, BT474 cells harboring shRNA against *c-Abl* (shAbl) or control shRNA of scrambled sequence (shCtrl) were treated with the Y211F or the scramble peptide (**Fig. 5C**). While Y211F peptide treatment significantly inhibited the growth of BT474/shLuc cells, the growth inhibition was partially rescued by knocking down c-Abl expression in BT474/shAbl cells. The reduced sensitivity of BT474/shABL cells to the Y211F peptide is consistent with the notion that c-Abl enhances cell growth at least in part through Y211 phosphorylation.

**Figure 6:**
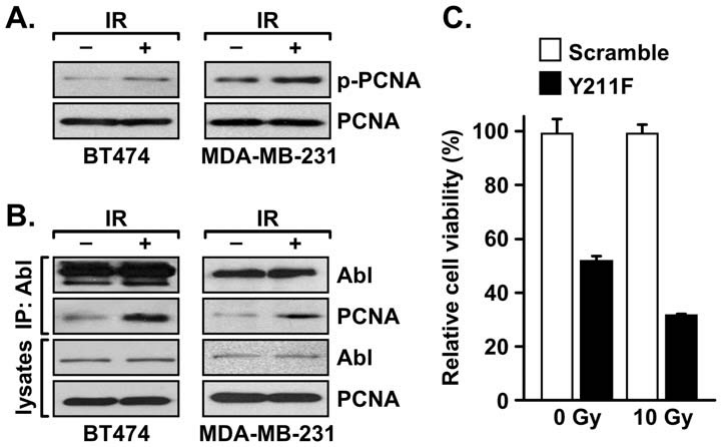
IR induced Y211 phosphorylation of PCNA and its interaction with c-Abl. **A.** BT474 and MAD-MB-231 cells were irradiated (10 Gy), and then incubated for 2 h. Cell lysates were immunoprecipitated by using the anti-phospho-Y211 antibody and then probed by western blotting with an anti-PCNA antibody. The input amount of PCNA in the lysates was also assessed (lower panel). **B.** IR induced the interaction between PCNA and c-Abl in breast cancer cells. Cells were treated as described in A, and then lysed. Endogenous c-Abl was immunoprecipitated by using an anti–c-Abl antibody, and the co-precipitated PCNA and c-Abl were detected by western analysis. **C.** BT474 cells were treated with the Y211F peptide or the scramble peptide (10 µM) for 12 h, then irradiated (10 Gy), followed by incubation for 36 h. Surviving cells were then assessed by MTT assay. Relative cell viability (*y* axis) denotes reduced cell survival compared with the scramble control, which is defined as 100%.

**Figure 7:**
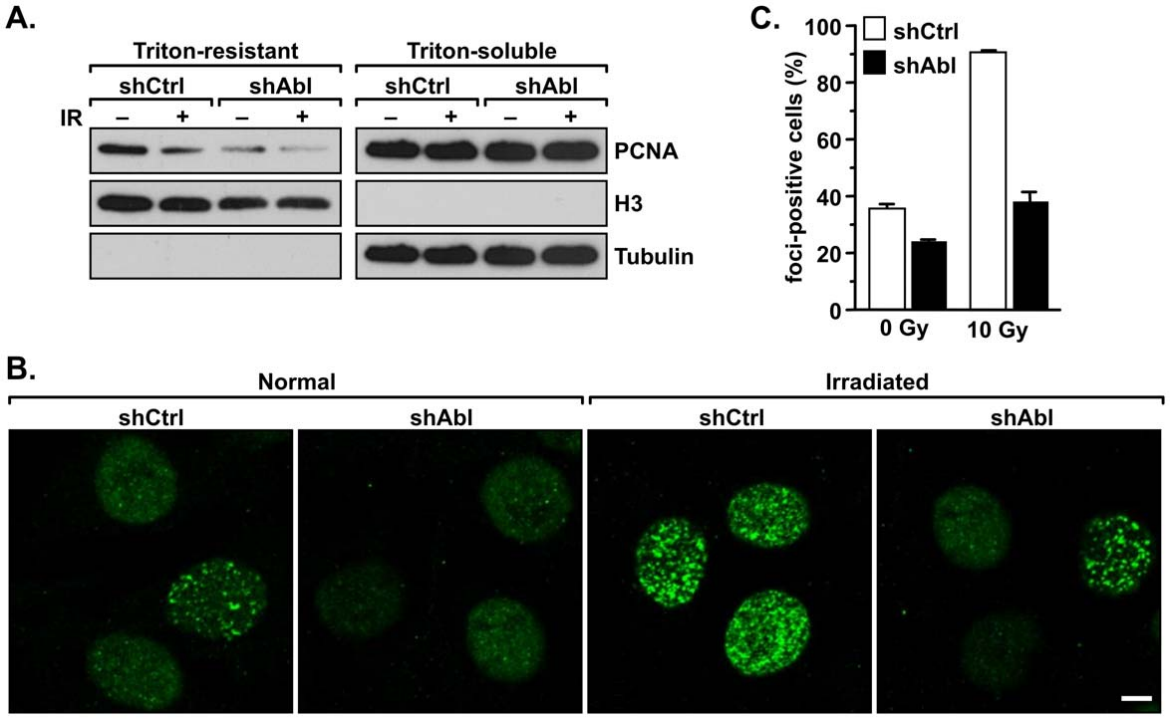
c-Abl enhances chromatin association and PCNA foci formation in response to DNA damage. **A.** MDA-MB-231/shAbl and MDA-MB-231/shCtrl cells were exposed to 10 Gy of IR. Following one hour of incubation, the cells were extracted with 0.5% of Triton X-100. Levels of PCNA in the soluble and insoluble fractions were examined by western analysis. Expression of α-tubulin and histone H3 was assessed as markers of the soluble and insoluble fractions, respectively. **B.** c-Abl is important in the formation of PCNA nuclear foci. MDA-MB-231/shAbl and MDA-MB-231/shCtrl cells were mock-treated or irradiated with 10 Gy of IR followed by incubation for one hour. Cells were then fixed with methanol and stained with an anti-PCNA antibody. **C.** To evaluate the number of foci-positive cells, five independent fields representing each treatment were counted. The experiment was repeated two times, and the results of the two trials were consistent.

To further define the mechanism by which c-Abl–PCNA interaction is regulated and to discern the functional consequences of this interaction, cells were treated with or without ionizing irradiation. We found that the levels of Y211 phosphorylation were increased by the treatment (**Fig. 6A**). Lysates of these cells were subjected to immunoprecipitation with an anti-c-Abl antibody. The levels of co-precipitated PCNA were then determined by western analysis (**Fig. 6B**). Corroborating the observation that c-Abl interacts with PCNA through Y211 phosphorylation (**Fig. 2**), and that Y211 phosphorylation was enhanced by IR treatment (**Fig. 5A**), the interaction between c-Abl and PCNA was also enhanced by IR treatment. These results taken together suggest that Y211 phosphorylation is an important signaling event in response to DNA-damage stresses in breast cancer cells. Indeed, blocking BT474 cells with the Y211F peptide, but not the control scramble peptide, sensitized cells to IR treatment (**Fig. 6C**). Corroborating these results, further experiments demonstrated that c-Abl plays a pivotal role in promoting the chromatin association of PCNA (**Fig. 7**). MDA-MB-231/shAbl and MDA-MB-231/shCtrl cells were extracted with Triton X-100 to isolate the chromatin-bound and chromatin-unbound fractions of PCNA. Down-regulation of c-Abl resulted in a significant decrease in chromatin-bound PCNA (**Fig. 7A**). IR treatment led to a further reduction of the chromatin-bound form of PCNA in both cell lines, with the MDA-MB-231/shAbl cells containing the lowest level of chromatin-associated PCNA. Interestingly, after IR treatment, the remaining chromatin-bound PCNA responded to DNA-damage stress by forming sub-nuclear foci (**Fig. 7B**). In the absence of stress, MDA-MB-231/shAbl cells had moderately fewer PCNA foci-positive cells than the MDA-MB-231/shCtrl control cells. Ionizing irradiation dramatically induced nuclear PCNA foci in MDA-MB-231/shCtrl cells. Such induction was not observed in MDA-MD-231/shAbl cells (**Fig. 7C**). Thus, depletion of c-Abl mitigates the PCNA-mediated DNA-damage response.

## Discussion

Y211 phosphorylation of PCNA represents a mechanism by which growth signaling regulates nuclear proliferation events [8], [20]. The post-translational modification was known to enhance PCNA stability on the chromatin. However, it was not known whether the phosphorylation event could also mediate PCNA regulatory signaling. In the current study, we demonstrated that phospho-Y211 PCNA forms a docking site for the non-receptor tyrosine kinase c-Abl and the resulting association with c-Abl enhances the level of chromatin-bound PCNA, in unperturbed as well as DNA damage-stressed cells. These results unveil the crosstalk between the DNA replication machinery and other nuclear signaling molecules to regulate cell growth. Given the versatile functions of PCNA in maintaining the integrity of the genome, further exploration of the underlying mechanisms of these functions can lead to a better understanding of how cell signaling in the nucleus shapes the dynamics of genomic stability in cancer cells.

Recently, He *et al.* reported that c-Abl interacted with PCNA through a putative PCNA-binding motif in the SH2 domain of c-Abl [22]. This proposed motif, with the amino acid sequence QRSI, is located at amino acid residues 160 to 163 of the c-Abl protein (NCBI reference sequence NP_005148.2). It was suggested by the authors that the QXXI signature is a PCNA-interacting motif. However, we tested mutant c-Abl in which that motif was mutated to ARSA, as used in that report, and found that the mutant appeared to have binding ability similar to that of the wild-type c-Abl (**Figure S2**), indicating that the PCNA did not bind to c-Abl through the QXXI motif in our experimental condition. This discrepancy could be due to different experimental conditions and/or the setting of the experiments. In the current study, the binding between the transfected c-Abl and the endogenous PCNA was assessed, while in the study by He *et al.* both proteins were ectopically expressed. It is noteworthy that although different binding sites are identified in these two studies, these findings are not necessarily mutually exclusive. For example, while the identified PCNA-binding motif of c-Abl may play a role in anti-apoptosis, interaction between Abl/SH2 with PCNA/phospho-Y211 can confer a signaling for growth advantage in cell proliferation. Further experiments using the corresponding c-Abl and PCNA mutants under unperturbed and stressed conditions should be able to test and characterize these two interaction modes.

Our previous study showed that expression of Y211-phosphorylated PCNA was associated with poor overall survival in breast cancer [20]. We recently reported that c-Abl expression is a frequent event in breast cancer [7]. The current study provides insight into the crosstalk between these two breast cancer markers. We reasoned that if phospho-Y211 PCNA has a major contribution to the growth-promoting function of c-Abl, down-regulation of Y211 phosphorylation should have a more significant impact on cells expressing c-Abl than cells lacking the c-Abl function. Indeed, Y211F peptide treatment conferred a more significant growth inhibition effect in cells expressing c-Abl than in the cells in which c-Abl was depleted (**Fig. 5C**). Such functional interaction between c-Abl and PCNA is also supported by the observation in **Figure 7** in which c-Abl depletion resulted in mitigated foci formation of PCNA in response to DNA damage. These results warrant further study to determine the role of Y211 phosphorylation of PCNA in response to growth stimulation and DNA-damaging stresses. Given that enhanced proliferation provides an essential growth advantage to cancer cells in primary tumors and in metastatic lesions and that cancer cells likely constitute the major proliferative compartment in a cancer patient, exploiting the proliferative function of PCNA for therapeutic gains has high potential to be effective in cancer treatment. We recently reported that phosphorylation of PCNA at Y211 is a promising treatment target in prostate cancer [23]. Our current results suggest that targeting phospho-Y211 PCNA could be an effective strategy in breast cancer treatment as well.

## Materials and Methods

### Cell culture, peptides, and antibodies

The breast cancer cell lines MDA-MB-231 and BT474 were purchased from American Type Culture Collection (ATCC) and grown in DMEM/F12 (1∶1) with 10% fetal bovine serum. The following peptides were synthesized at the Keck Peptide-synthesis Facility of Yale University: TAT-conjugated peptides including the wild-type Y211 non-phosphorylated peptide (WT; Figure 2E) (Ac-CGRKKRRQRRRGTFALRYLNFFTK-CONH_2_), the wild-type Y211-phosphorylated peptide (Y211-P; Figure 2E) (Ac-CGRKKRRQRRRGTFALRYpLNFFTK-CONH_2_), the Y211F peptide (Ac-CGRKKRRQRRRGTFALRFLNFFTK-CONH_2_), the scrambled control peptide (Ac-CGRKKRRQRRRGFLFTNKLFRTAF-CONH_2_), the purified N-terminally FAM-conjugated TAT-Y211F peptide (FAM-TAT-Y211F; Figure 2B), and the Y211F peptide without TAT sequence (FAM-Y211F; Figure 2B) were. The following antibodies used in this study were purchased from a variety of manufacturers: α-tubulin (Sigma; St. Louis, MO); c-Abl (BD Pharmingen; San Jose, CA), Histone H3, PCNA (Santa Cruz; Santa Cruz, CA), phospho-Y211 PCNA (Bethyl; Montgomery, TX).

### Proliferation analysis

Cell proliferation was assessed by using a colorimetric BrdU proliferation kit according to the manufacturer's instructions (Roche, Indianapolis, IN; Cat. No. 11647229001). Briefly, 1000 to 3000 cells per well were plated in 96-well plates in triplicate. The experiments were repeated at least three times. Cells treated with the peptides were labeled with BrdU for 3 to 4 h. The genomic DNA was fixed and denatured, and then incubated with peroxidase-conjugated anti-BrdU antibody for 90 min. A substrate for the conjugated peroxidase was then added and the reaction product was quantified by measuring the absorbance. The results were then normalized by the number of total viable cells, which was determined by a side-by-side cell-viability assay, as described above.

### Cell-cycle analyses by flow cytometry

Cells were harvested by trypsin, washed with PBS, and then fixed in 70% ethanol. The fixed cells were stained with 25 µg/ml propidium iodide (Sigma; St. Louis, MO) in the presence of 1 µg/ml RNase (Sigma; St. Louis, MO). For fluorescence-activated cell sorting (FACS) analysis, data were collected using a FACSCalibur flow cytometer and analyzed using the software ModFit (Verity; Topsham, ME). The cell-cycle distribution was evaluated by counting >10,000 cells per sample.

### Immunoprecipitation and western blotting analysis

Cells were lysed by incubation with NETN buffer (150 mM NaCl, 1 mM EDTA pH 8.0, 20 mM Tris pH 8.0, 0.5% NP-40, 25 mM NaF, 2 mM Na_3_VO_4_, 20 µl/ml aprotinin (Sigma), 0.1 M PMSF). For western analysis, the lysates were separated in acrylamide gels, transferred to a PVDF membrane (Bio-Rad; Hercules, CA), and probed with the indicated antibodies. Bands were visualized by a chemiluminescence-based detection method (Fisher/Pierce; Rockford, IL) that used horseradish peroxidase-conjugated secondary antibodies. For immunoprecipitation, 1 to 2 mg of protein was used for each reaction. Proteins were incubated with the antibody at 4°C overnight. Protein G agarose was then added to precipitate the antibody-protein complex. The beads were then washed four times with NETN buffer. The immunocomplexes were then released by boiling in 2× loading buffer followed by western blotting analysis, as described.

### Immunofluorescence confocal microscopy

Cells were fixed and then permeabilized by methanol and 0.5% Triton X-100 for 10 minutes at room temperature. After four washes with PBS, the cells were blocked with 10% normal goat serum for 1 hour at room temperature, and then immunostained with primary antibody against PCNA (1∶200 dilution in PBS with 0.2% BSA) overnight at 4°C. After three washes with PBS, the FITC-conjugated secondary antibody was applied for 45 minutes at room temperature. Images were captured with a Zeiss laser scanning confocal microscope (LSM510). For localization analysis of the FAM-labeled peptides, cells were treated with the peptide at 7.5 µM for 30 min. Treated cells were then washed twice with PBS, and fixed with 4% paraformaldehyde. Peptide localization was visualized by fluorescence confocal microscopy as described above.

### Statistical analysis

Data from each assay were expressed as means ± SD (n = 3). Statistical differences between two groups were determined by the Student's t-test. *P*<0.05 was considered significantly different.

## Supporting Information

Figure S1 **Expression of FLAG-PCNA in HEK293T cells.** Cells were transfected with FLAG-PCNA (wild-type, Y211F) or the empty vector (pcDNA3). The lysates of the transfected cells were analyzed by western blotting using an anti-PCNA antibody (Santa Cruz; Santa Cruz, CA). Both the endogenous (endo PCNA) and the FLAG-tagged ectopic PCNA (Flag-PCNA) were shown as indicated by the arrows.
(TIF)

Figure S2 **Assess the putative PCNA-binding motif of c-Abl.** The c-Abl mutant c-Abl/QI-AA had no effect on the interaction between PCNA and c-Abl. HEK293T cells were transfected with the cDNA of the Myc-tagged wild-type or QI-AA mutant of c-Abl or the control vector. Cell lysates were then immunoprecipitated with an anti-Myc antibody. The levels of co-precipitated endogenous PCNA were determined by western analysis using an anti-PCNA antibody.
(TIF)
