# Supplementary figures and images for: Interaction of Proliferation Cell Nuclear Antigen (PCNA) with c-Abl in Cell Proliferation and Response to DNA Damages in Breast Cancer
Source: PLoS One. 2012 Jan 4;7(1):e29416. doi: 10.1371/journal.pone.0029416 (PMC3251568; doi:10.1371/journal.pone.0029416)

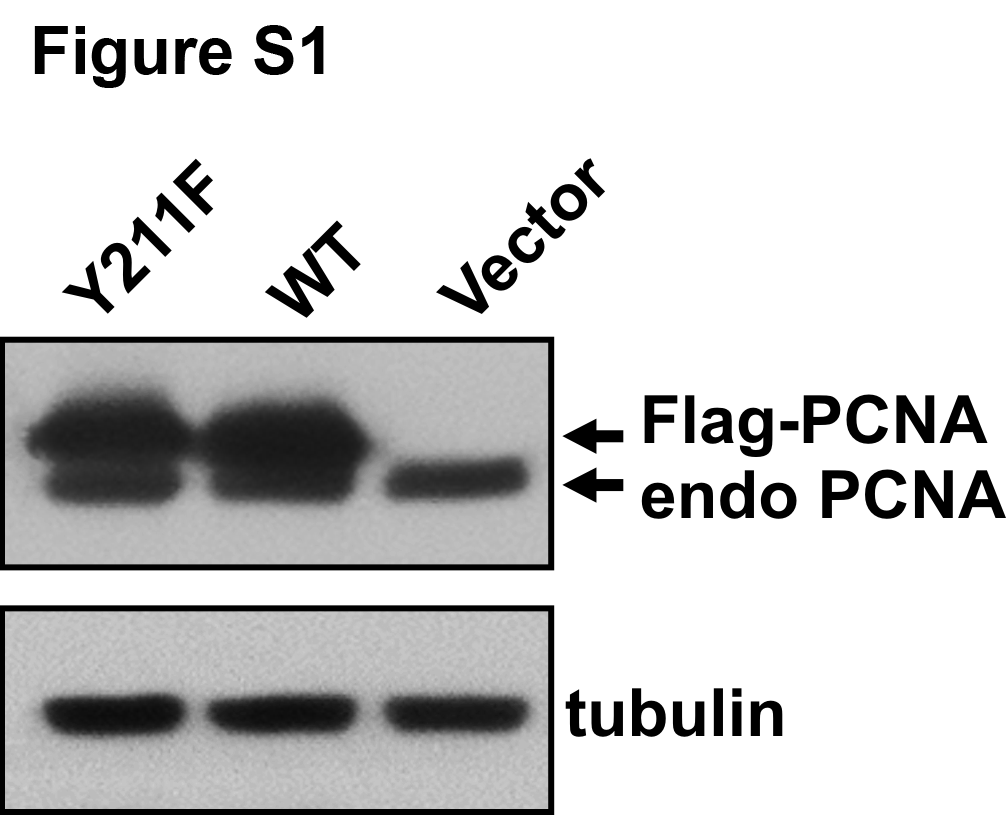

Supplement: Figure S1 — Expression of FLAG-PCNA in HEK293T cells. Cells were transfected with FLAG-PCNA (wild-type, Y211F) or the empty vector (pcDNA3). The lysates of the transfected cells were analyzed by western blotting using an anti-PCNA antibody (Santa Cruz; Santa Cruz, CA). Both the endogenous (endo PCNA) and the FLAG-tagged ectopic PCNA (Flag-PCNA) were shown as indicated by the arrows. (TIF) [file pone.0029416.s001.tif]

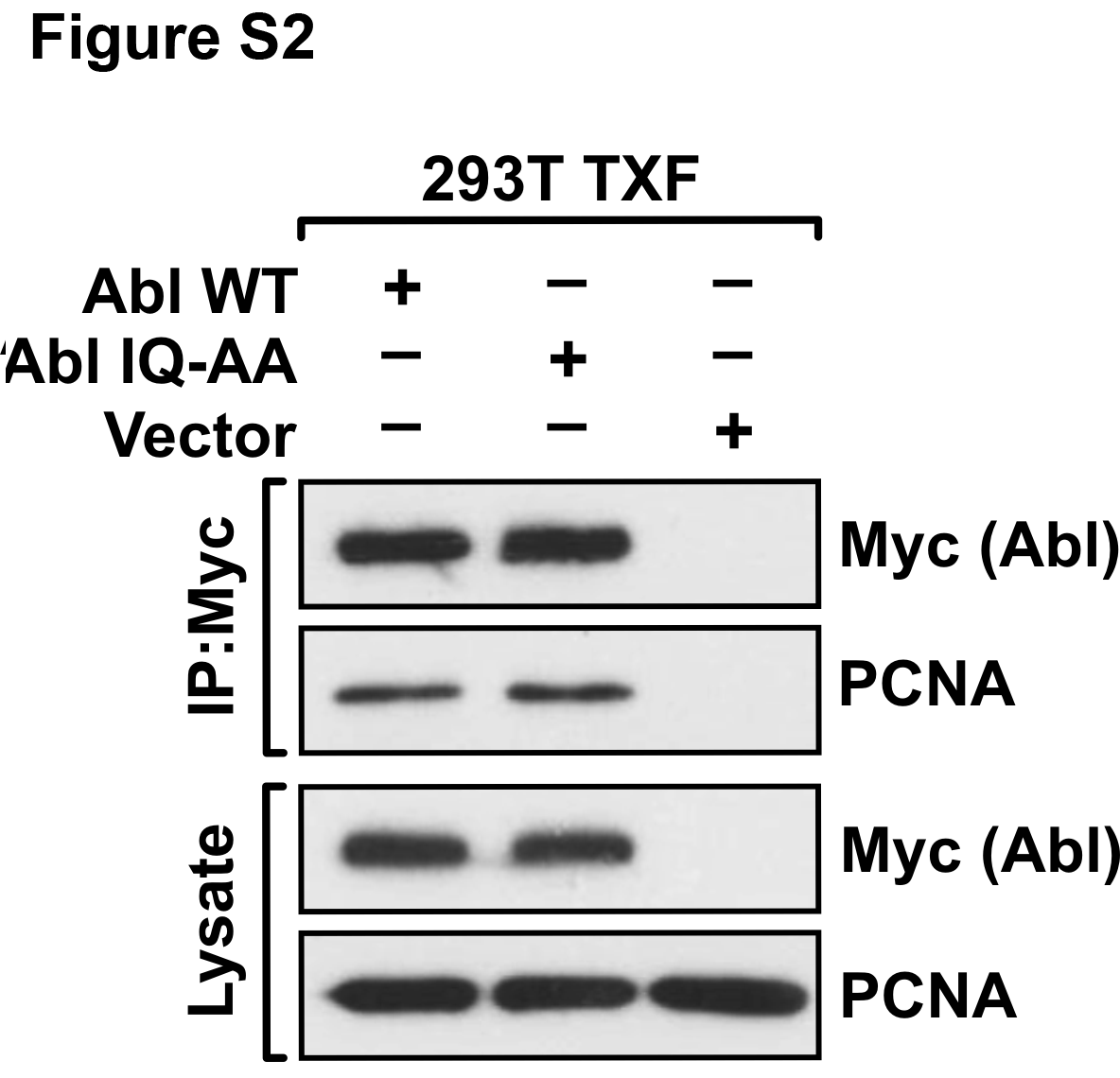

Supplement: Figure S2 — Assess the putative PCNA-binding motif of c-Abl. The c-Abl mutant c-Abl/QI-AA had no effect on the interaction between PCNA and c-Abl. HEK293T cells were transfected with the cDNA of the Myc-tagged wild-type or QI-AA mutant of c-Abl or the control vector. Cell lysates were then immunoprecipitated with an anti-Myc antibody. The levels of co-precipitated endogenous PCNA were determined by western analysis using an anti-PCNA antibody. (TIF) [file pone.0029416.s002.tif]
